# Influence of Climate Warming on Arctic Mammals? New Insights from Ancient DNA Studies of the Collared Lemming *Dicrostonyx torquatus*

**DOI:** 10.1371/journal.pone.0010447

**Published:** 2010-05-27

**Authors:** Stefan Prost, Nickolay Smirnov, Vadim B. Fedorov, Robert S. Sommer, Mathias Stiller, Doris Nagel, Michael Knapp, Michael Hofreiter

**Affiliations:** 1 Research Group Molecular Ecology, Max-Planck Institute for Evolutionary Anthropology, Leipzig, Germany; 2 Department of Paleontology, University of Vienna, Vienna, Austria; 3 Institute of Plant and Animal Ecology, Russian Academy of Sciences, Yekaterinburg, Russia; 4 Institute of Arctic Biology, University of Alaska Fairbanks, Fairbanks, Alaska, United States of America; 5 Ecology Centre, Christian-Albrechts-University of Kiel, Kiel, Germany; Natural History Museum of Denmark, Denmark

## Abstract

**Background:**

Global temperature increased by approximately half a degree (Celsius) within the last 150 years. Even this moderate warming had major impacts on Earth's ecological and biological systems, especially in the Arctic where the magnitude of abiotic changes even exceeds those in temperate and tropical biomes. Therefore, understanding the biological consequences of climate change on high latitudes is of critical importance for future conservation of the species living in this habitat. The past 25,000 years can be used as a model for such changes, as they were marked by prominent climatic changes that influenced geographical distribution, demographic history and pattern of genetic variation of many extant species. We sequenced ancient and modern DNA of the collared lemming (*Dicrostonyx torquatus*), which is a key species of the arctic biota, from a single site (Pymva Shor, Northern Pre Urals, Russia) to see if climate warming events after the Last Glacial Maximum had detectable effects on the genetic variation of this arctic rodent species, which is strongly associated with a cold and dry climate.

**Results:**

Using three dimensional network reconstructions we found a dramatic decline in genetic diversity following the LGM. Model-based approaches such as Approximate Bayesian Computation and Markov Chain Monte Carlo based Bayesian inference show that there is evidence for a population decline in the collared lemming following the LGM, with the population size dropping to a minimum during the Greenland Interstadial 1 (Bølling/Allerød) warming phase at 14.5 kyrs BP.

**Conclusion:**

Our results show that previous climate warming events had a strong influence on genetic diversity and population size of collared lemmings. Due to its already severely compromised genetic diversity a similar population reduction as a result of the predicted future climate change could completely abolish the remaining genetic diversity in this population. Local population extinctions of collared lemmings would have severe effects on the arctic ecosystem, as collared lemmings are a key species in the trophic interactions and ecosystem processes in the Arctic.

## Introduction

Both in public perception and the scientific literature, climate change has become an important topic due to its presumed impact on Earth's ecological and biological systems. Changes in climate such as temperature increase may result in a loss of biodiversity, higher dispersion of diseases, poleward shifts of species ranges, shifts in phenological events (e.g. reproduction, blooming) and even species extinction [1]–[7]. According to the IPCC the global average temperature is likely to increase by between 1.4 and 5.8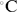 over the period from 1990 to 2100 [8]. Many studies investigated the biological impacts of recent global warming (e.g. [1], [2]) concluding that even the moderate warming of the global temperature by half a degree to date already had major effects [2]. The magnitude of abiotic changes in the Arctic is even larger than in temperate and tropical biomes [9]–[11], further emphasizing the importance of a proper knowledge of the biological effects of climate change, especially in the Arctic [11]. Yet the biological consequences of climate change on high latitudes remain relatively underreported [11] and surprisingly little is known about the effects of climate change on the genetic diversity of species [12], although a proper understanding of the effects of climate change on species on the population level is crucial for assessment and prediction of future scenarios.

An excellent approach to improve our knowledge about climate change and its implications is to study the past. The Quaternary period (the past 2.6 My) was marked by cycles of glacial-interglacial changes [13], [14] and thus can be used as a model system to study past demographic changes correlating with climate change. The period following the Last Glacial Maximum (LGM, 25.0-18.0 cal. kyrs BP) was characterized by two major climate warming events: the Greenland Interstadial 1 (Bølling/Allerød warming phase at 14.5 kyrs cal. BP) and the transition from the late Pleistocene to the Holocene starting at 11.5 cal. kyrs BP, when a drastic temperature increase occurred within only a few decades [15]–[17]. These climate warming events resulted in widespread vegetation shifts [18] and faunal extinctions in the Arctic [19].

Ancient DNA provides an outstanding tool to study past population dynamics as it allows a survey of genetic variation in populations over hundreds to thousands of generations and enables the reconstruction of temporal demographic history on a reasonable time scale. In order to reveal biotic responses, reconstructed demographic changes are aligned with the chronology of climatic events inferred from independent lines of evidence. This “phylochronological” approach [12] is a powerful tool to study temporal demographic changes using serial ancient DNA sampling. In one of the first studies, Hadly et al. 2004 [12] analyzed temporal changes of genetic diversity in a single population of the pocket gopher over 2,500 yrs.

The collared lemmings (*Dicrostonyx sp.*), the northernmost genus of rodents and key species of the arctic community, evolved in dry landscapes of eastern Siberia and were characteristically associated with the dry and cold environment of the tundra steppe during the Pleistocene [20]–[22]. In order to study the effects of the climate warming events that occurred at 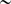
14.7 and 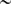
11.5 cal. kyrs BP on Arctic adapted species, we sampled fossil and modern bones of the collared lemming, *Dicrostonyx torquatus*, from a single site (Pymva-Shor) in the northern Pre-Urals, for ancient DNA analyses. The layers of this site are dated to 21,910
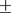
250 (L6 low), 13,090
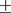
60 (L6 up), 10,000
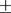
250 (L4) yrs BP and modern (surface; calibrated dates are provided in Table S1), thus representing sampling before, during and after climatic events that, based on the ecological preferences of this arctic species should have generated demographic changes.

The paleontological record from the region surrounding and including Pymva Shor shows a decrease in the numbers of fossil remains after the LGM (Figure 4 in [23]), that continues for thousands of years and results in the complete absence of the fossil record for this species around the Holocene thermal optimum (8 to 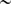
4.5 kyrs).

So far, little is known about species responses to climate change in the Arctic. Our study aims to fill this gap and improve the understanding of past population responses of arctic species to climate change. In the case of the collared lemmings this is particularly important since demographic changes in this species have strong consequences for trophic interactions and ecosystem processes in the Arctic [11], [24]–[30].

## Results

We extracted mitochondrial DNA from 54 ancient and 10 modern collared lemmings from Pymva Shor (Northern Ural, Russia) and 13 ancient samples from Yangana-Pe-4 (Northern Ural, Russia). From all extracts, we successfully amplified and sequenced 282bp of the cytochrome B (CytB) gene and 426bp of the control region (CR). Surprisingly we found the control region to show less variability per nucleotide position than cytochrome b. Although this is an unusual result, as the control region usually comprises the most variable part of the mitochondrial genome, this observation is not unprecedented. It is consistent with observations in modern collared lemmings [31] and birds [32], but in contrast to mtDNA variation in other rodents [33].

### Inference of temporal demographic changes

**Figure 1:**
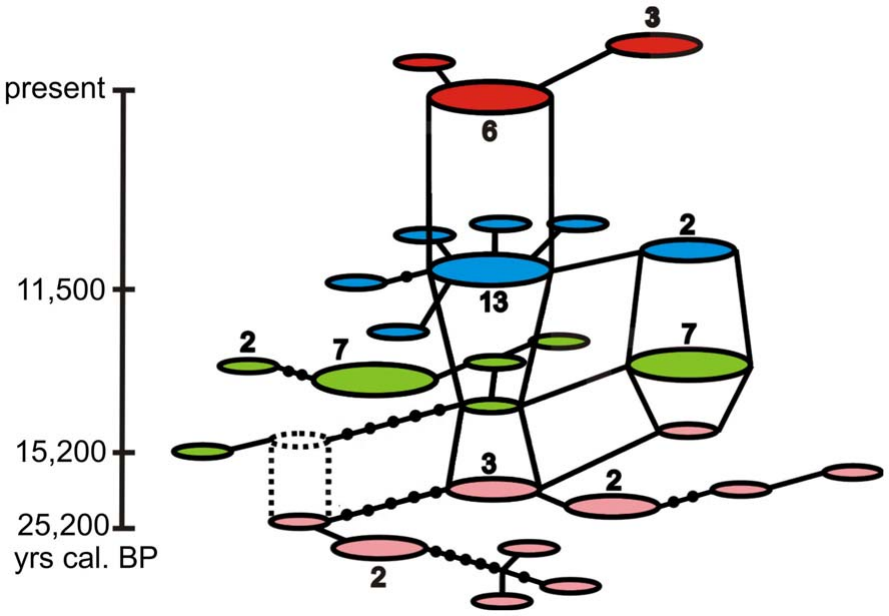
Three dimensional statistical parsimony network. The network is structured in four layers comprising haplotype samplings at different age (rose: 25,200 cal. yrs BP, green: 15,200 cal. yrs BP, blue: 11,500 cal. yrs BP, red: modern). Each circle represents a separate haplotype. The lines between circles show the number of substitutions separating two haplotypes. If the haplotypes are separated by more than one substitution the number of cross lines plus one refers to the actual number of substitutions. Dashed circles and lines indicate an unsampled intermediate haplotype sampled at the previous time-point. Numbers above or below circles indicate the actual number of individuals belonging to this haplotype. Only numbers higher than one are displayed.

First, we constructed a temporal network, to display the haplotype composition through time (Figure 1). This analysis shows that genetic diversity decreased drastically over time, with the highest amount of genetic variation being present around the LGM and the lowest in the modern population. Nearly all distinct haplotypes (different by more than one substitution from the next nearest haplotype in the network) were lost between 15,200 and 11,500 cal. yrs BP. The network also reveals two major haplotypes, which were present in all, or in all but the modern population. The number of haplotypes decreases from ten at 25,200 cal. yrs BP to seven at 15,200 and 11,500 cal. yrs BP and further after 11,500 cal. yrs BP to only three haplotypes in the present (Figure 1 and Table 1).

**Table 1:** Statistics summarizing the data.

Age (cal. yrs BP)	Seg.Sites	Haplotypes	Pairw. Diff.	Nucleo. Div.	Hapl. Div.
**Cytochrome B - 282bp**					
Modern	1	2	0.467	0.00165	0.467
11.500	6	6	0.689	0.00244	0.516
15.200	8	6	1.868	0.00663	0.737
25.200	12	7	3.736	0.01325	0.901
**Control Region - 426bp**					
Modern	1	2	0.200	0.00047	0.200
11.500	1	2	0.100	0.00023	0.100
15.200	5	4	0.679	0.00159	0.437
25.200	6	8	2.451	0.00575	0.824
**Combined - 708bp**					
Modern	2	3	0.667	0.00094	0.600
11.500	7	7	0.789	0.00112	0.584
15.200	13	7	2.547	0.00360	0.774
25.200	18	10	6.187	0.00874	0.945

We estimated different summary statistics for the two genes separately and for the combined region. The number of segregating sites directly reflects the genetic variability and therefore the effective population size. Additionally we provided frequency based statistics such as average pairwise sequence difference. Interestingly we found higher variation in cytochrome b gene than in the control region, which is consistent with observations in modern collared lemmings [31] but in contrast to mtDNA variation in other rodents [33]. (Seg.Sites…segregating sites, Haplotypes…number of haplotypes, Pairw. Diff…average pairwise sequence difference, Nucleo. Div…nucleotide diversity, Hapl.Div…haplotype diversity).

We tested the possibility that sampling bias resulted in the observed pattern by simulating a temporal data set under a constant population size model. Using average pairwise sequence difference as a summary statistic reflecting the genetic diversity within sample points we found little support for the two youngest sample points (modern and 11,500 cal. yrs BP, Figure S1) under a constant size model with P-values of 0.00988 and 0.00654 respectively, but higher support for the most diverse and oldest sample points (15,200 and 25,200 cal. yrs BP, Figure S1) with P-values of 0.18757 and 0.22578 respectively. Thus we were able to reject the hypothesis that the low genetic diversity observed at the two youngest data points could be obtained by chance.

**Figure 2:**
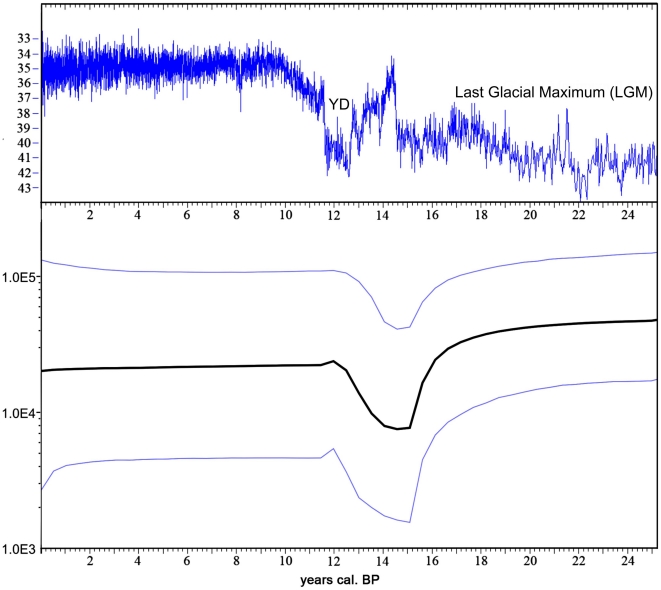
Climate history as derived from the GISP2 ice-core (upper panel) and demographic history of the collared lemming *Dicrostonyx torquatus* (lower panel) during the last 25,200 yrs. Upper panel: oxygen isotope (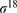
O) deviations in ‰ (y-axis). The climate graph was calculated using the program CalPal [85]. Lower panel: Bayesian Skyline Plot reconstruction of the temporal demographic history of *Dicrostonyx torquatus*. The thick black line is the median estimate of the fNe (y-axis) over time and the blue lines indicate the 95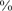 highest posterior density intervals (HPD).

Next, we calculated a Bayesian Skyline Plot (BSP) [34] using MCMC based Bayesian inference as implemented in the Software BEAST 1.4.8. [35]. Female effective population sizes at different time points were estimated from the lineage coalescent rate through time. In order to reduce noise introduced by outliers in the data set, we summarized neighboring coalescent intervals into groups and analyzed the data with varying numbers of groups. We analyzed runs with 6, 10 and 12 groups. The BSP (Figure 2) shows a decrease in the female effective population size (fNe) starting around the end of the LGM. This fNe decline reaches its maximum between 14,000 and 15,000 cal. yrs BP (assuming a generation time of one year). After a short recovery phase there is another inflection point at around 12,000 cal. yrs BP after which the population size stays approximately constant. However, the highest posterior density (HPD) intervals broaden between 11,500 cal. yrs BP and the present. This population decline followed by a subsequent increase was observed in all analyses independent of the number of coalescence interval groups (i.e 6, 10 or 12). Anyway, the signal magnitude changed, with the strongest signal under a 12 group prior and the weakest signal under a 6 group prior. This result is expected, since fewer groups will cause a strong averaging of population sizes from coalescent intervals preceding and following the bottleneck (data not shown). We further used Tracer 1.4 [36] to compare the results of the BSP reconstruction with a simple constant population model. The calculated log10 Bayes factor of 1.0 suggests a substantial to strong support for the BSP reconstruction [37].

The mutation rate was estimated at 0.06 (ABC: fragmentation model) and 0.085 (ABC: bottleneck model) to 0.09 mutations/site/myrs (MCMC method). To confirm that the mutation rate was not only depending on the sample age (i.e if the sequence data was uninformative) we repeated the BSP analyses with 20 datasets in which sequence data and sample age were randomly shuffled and found that the estimate for our original data set clearly falls outside the range of the estimates from the randomly shuffled datasets indicating that there is sufficient variation in the data to reliably estimate the mutation rate. The marginal densities of the test runs are shown in Figure S2 in the online supplementary material.

### Assessing possible gene flow between locations

**Figure 3:**
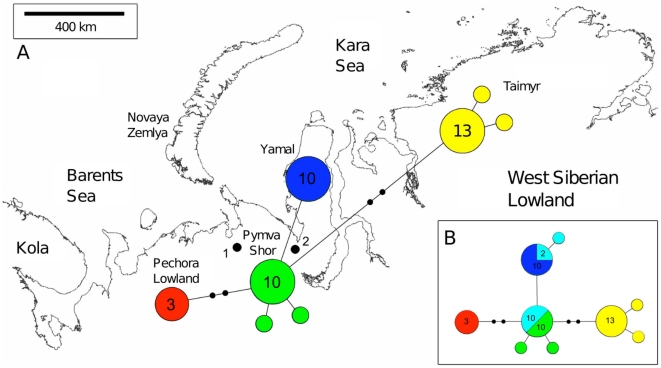
Statistical parsimony networks showing phylogenetic relationships among mtDNA haplotypes from different regions adjacent to Pymva Shor. Main panel: Statistical parsimony network showing phylogenetic relationships among modern mtDNA haplotypes from different regions adjacent to Pymva Shor. Lower right: Statistical parsimony network showing relationships among modern mtDNA haplotypes from different populations adjacent to Pymva Shor including ancient DNA sequences (younger than 1,000 years) from Yangana-Pe-4 (1: Pymva Shor, 2: Yangana-Pe-4). Circles represent haplotypes and colors refer to locality sampled (red: Pechora, green: Pymva Shor, blue: Yamal, yellow: NW-Taimyr and cyan: Yangana-Pe-4). Numbers in the circles refer to the actual number of individuals carrying a haplotype. Only numbers bigger than one are shown.

We reconstructed additional statistical parsimony networks including just modern (Figure 3A) and modern and up to 1,000 yrs old samples from Yangana-Pe-4 (Figure 3B) to address the question of whether the data bears signs of modern gene flow and if so to what extend (Figure 3). The modern data shows a restriction of all haplotypes to just a single geographic location, a phylogeographic structure that implies that no recent gene flow occurred between the regions investigated. However, we found the most prominent haplotypes from both PS and Yamal in the 1,000 year old samples from Yangana-Pe-4 (which is situated between Yamal and PS). In total, the samples from Yangana-Pe-4 revealed three haplotypes. Two haplotypes are identical or closely related to the Yamal haplotype (comprising 3 sequences). All other sequences (10) belong to the major haplotype within PS, indicating historical gene flow from both PS and Yamal into Yangana-Pe-4 or vice versa. Furthermore, four unique, slightly divergent haplotypes (each differing by one mutations from the most common one) were found in the sampling from PS dating to 11,500 cal. yrs BP, which could be interpreted as further evidence of gene flow (see discussion).

**Figure 4:**
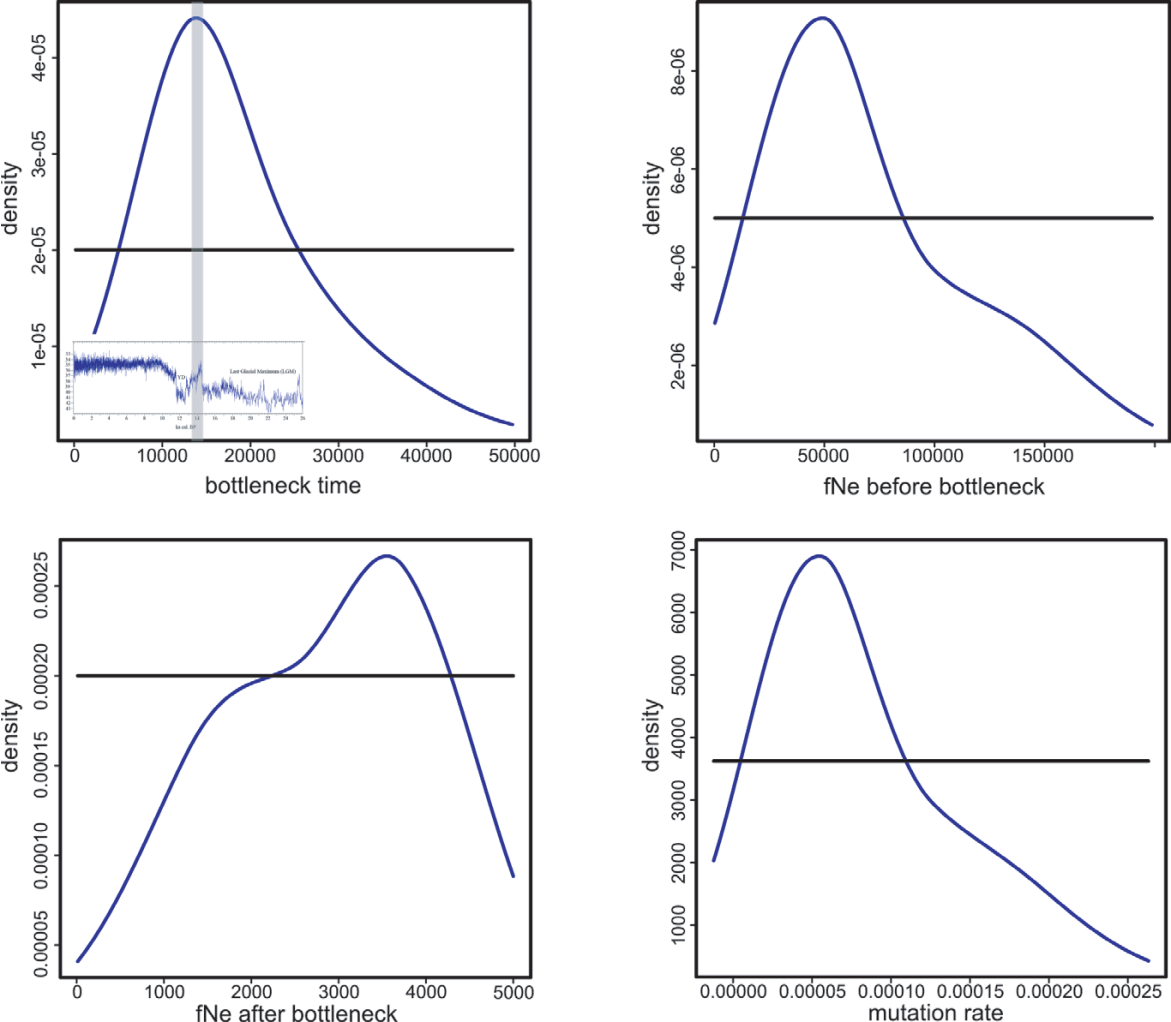
Posterior probability distributions of different model parameters of the ABC analysis. Upper left: Density curve of the bottleneck time. The grey bar shows the 5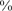 quantile of the highest posterior density, which matches to the first major temperature increase at the Pleistocene-Holocene transition (as indicated in the small climate reconstruction). Upper right: Density curve of the female effective population size prior to the bottleneck. Lower left: Density curve of the female effective population size after the bottleneck. Lower right: Density curve of the mutation rate. The prior distributions are represented as black lines in all the plots.

In order to verify these results we used the approximate Bayesian computation (ABC) approach [38] to test whether a closed single-population-bottleneck model (no gene flow), an open population-bottleneck model (gene flow following the period of minimum population size) or a constant-population-size model provided the best fit for our data. The posterior probability distribution for the timing of the bottleneck, fNe after and before the bottleneck and the estimated mutation rate of the closed population-bottleneck model can be seen in Table 2. The posterior probability distributions of both bottleneck models show clear peaks confirming that the genetic data contains sufficient information and that the results are not driven by the priors (Figure 4 for the closed population-bottleneck model or Figure S3 for the open population-bottleneck model). The open population-bottleneck model was supported more strongly (55.8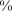) in the model comparison method applying a rejection and regression step [39] than the closed population bottleneck model (44.2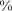) and the constant-population-size model (0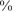), weakly suggesting ancient gene flow between PS and neighboring regions (Figure S4). Histograms of the 6,000 closest estimates can bee seen in Figure S5 showing that the specified models indeed fit the observed data.

**Table 2:** Parameter - Mode and Quantiles (5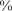, 25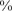, 95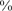).

Parameter	Mode	Quantile					
		5 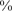		25 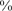		95 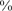	
		LLim	ULim	LLim	ULim	LLim	ULim
Bottleneck timing	13,998.5	13,392.9	14,571.4	10785.0	16,813.8	146.0	40,448.7
fNe before the Bn	28,331.1	25,535.9	31,170.0	14,508.0	42,918.6	269.87	17,8071.2
fNe after the Bn	3,690.6	3,591.2	3,785.5	3,117.1	4,114.8	122.9	5,053.8
Modern fNe	11,090.3	10,221.9	11,969.6	6,589.1	1,5432	27.09587	46,428.6
mutation rate	5.60e-05	5.22e-05	5.98e-05	3.72e-05	7.55e-05	7.09e-06	0.000242
Growth rate	−1.84e-04	−1.94e-04	−1.72e-04	−2.37e-04	−1.29e-04	−0.000753	0.000336
fNe scaling factor	10.2	9.2	11.5	4.33	16.4	0.08	134.2

Mode and quantiles (5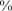, 25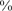, 95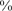) of four model parameters of the ABC analysis. Lower (LLim) and upper (ULim) limits of the respective quantile are provided for the bottleneck timing, the female effective population size after (fNe after the Bn) and before (fNe before the Bn) the bottleneck and the modern effective population size along with the mutation rate estimated (mutations/locus/generation), growth rate and the new fNE scaling factor (to simulate a population reduction).

## Discussion

During the Late Pleistocene the collared lemming, a key species of the arctic community, inhabited large parts of the Holarctic, especially in northern Eurasia [40]. Today, it is present only in the Arctic with a nearly circumpolar distribution (e.g. [40]). It is restricted to dry and treeless tundra and is the only rodent genus that inhabits the polar desert of the northernmost parts of the Eurasian and Canadian Arctic [41], [42]. Collared lemmings show a range of unique features that clearly indicate their adaptation to the Arctic environment. In winter they develop a white pelage and enlarged forefeet claws which allow them to dig through compacted snow [43]. Availability of Arctic shrubs and herbs such as those found in dry tundra habitats, are major ecological requirements of collared lemmings [41], [42], [44]. They provide both food and good burrowing conditions. In times of warm and humid climates in the Siberian lowlands, the dry Arctic tundra habitats preferred by collared lemmings shrank and became replaced by southern plant communities [27]. The fossil record shows that during the glacial advances the distribution range of collared lemmings expanded thousands of kilometers to the south while it contracted to the north during warm interglacials [45], [46]. In addition, to the paleontological record, genetic data from extant populations of collared lemmings in the Eurasian Arctic implies that this species underwent a continent-wide population contraction, possibly during one of the warm interglacials, followed by demographic expansion (e.g. [40], [47], [48]). Modern DNA sequences of *Dicrostonyx torquatus* show a restriction of haplotypes to limited geographic regions [40]. Furthermore, within these regional phylogeographical groups, low nucleotide and haplotype diversity and star-like haplotype phylogenies were found. These findings suggest regional bottleneck events, probably during the Holocene warming events [40].

Using ancient DNA sequences, we found genetic signs of population decline in the collared lemming in the period following the LGM. The reduction of the effective population size showed its maximum at the Greenland Interstadial 1 (Bølling/Allerød) warming phase at 14,500 yrs BP, which is congruent with predictions based on the ecological preferences of this Arctic species. Thus, the high genetic variation during the LGM most likely reflects a large and stable effective population size during the dry and cold climatic conditions of the Polar Ural and adjacent regions during this time [49], which are optimal for collared lemmings. The temporal network constructed from ancient and modern DNA sequences from a single site (Pymva Shor) in the northern Pre-Urals (Figure 1) shows a marked decline in genetic variation over time. Nearly all distinct haplotypes (differing by more than one substitution from the nearest haplotype in the network) were lost by 11,500 cal. yrs BP. Our simulations suggest that given the number of samples analyzed, it is unlikely that the low diversity observed at 11,500 cal. yrs BP and the present was obtained by chance and support the notion of a decline in genetic diversity over time. Such a pronounced demographic reduction is consistent with the existence of a bottleneck during that time. Model-based temporal demographic reconstructions further support our hypothesis of a bottleneck event after the LGM. Both MCMC based Bayesian inference and ABC simulations indicate that the population decline corresponds to the abrupt temperature increase around 14,700 cal. yrs BP during the Greenland Interstadial 1 (Bølling/Allerød) warming event. The fossil record of the region surrounding Pymva Shor showed the highest number of collared lemmings within the coldest and most arid periods (40,000 to 45,000 yrs and 21,000 to 18,000 yrs BP) [23]. In layers dating to warmer climatic periods, the proportions of collared lemming fossils decreased. During the Holocene thermal optimum between 8,000 and 4,500 yrs BP fossils of the collared lemmings were absent and these layers are dominated by taiga forest species such as the red vole (most prominent remains) [23]. The decrease in proportion of collared lemming remains along with the temperature increase and the faunal changeover from cold tundra adapted species to taiga species indicate significant effects of climate change on the biota during the Holocene-Pleistocene transition and the Holocene thermal optimum. Furthermore, paleontological and genetic data both indicate a severe population decline in *Dicrostonyx torquatus* correlating with the abrupt climate change after the LGM. Based on the ecological requirements of the collared lemming and the fossil record one would expect a population decline at 11,500 cal. yrs BP (Holocene-Pleistocene transition) at the beginning of the warm period of the Holocene. However, such a decline was not detectable in the MCMC analysis. Since all divergent haplotypes went extinct before 11,500 generations ago we suggest that most of the power to detect a potential population decline using DNA sequences was lost due to low genetic variation in the modern population. This suggestion is further supported by larger highest posterior density intervals (HPD) in the Bayesian Skyline Plot after 11,500 cal. yrs BP. To overcome this lack of information further sampling between 11,500 cal. yrs BP and the present would be necessary in order to obtain a better resolution for the demographic events between these two points in time. In addition given the already small number of haplotypes observed at 11,500 cal. yrs BP, extensive sampling would be necessary to obtain a sufficiently strong signal in the demographic reconstructions. This would be difficult given the low numbers of available samples and the lack of layers between 8,000 and the present in Pymva Shor. It is worthwhile to note, though, that the number of haplotypes detected declines from seven at 11,500 cal. yrs BP to only three in the modern population. In combination with the lack of collared lemming fossils during the Holocene climate optimum, these data strongly suggest that a further population size decrease took place after the end of the Pleistocene.

We consider it unlikely that cyclic population density fluctuations that occur at multiple-year intervals generated the observed decline in genetic variation over thousands of years. Population cycles represent more or less regular annual density fluctuations that are reported for small mammals especially voles and lemmings at northern latitudes (e.g. [29], [50]–[54]). Although the causality of these population cycles is under debate, some evidence suggests climate as an important factor [29], [54]. Low phases of population cycles represent repeating bottlenecks and are expected to decrease genetic variation due to genetic drift within populations. However, it was suggested that in lemmings, gene flow increases following a population decline and eliminates, or at least reduces the effect of genetic drift on intrapopulation diversity [55]–[59]. Compiling data on mtDNA polymorphism for 72 lemming populations from 5 species provided no evidence for the hypothesis that a decrease in genetic diversity results from cyclic density fluctuations [55].

Another crucial factor to infer from temporal data is gene flow. The four singleton haplotypes observed at PS at 11,500 cal. yrs BP could indicate possible migration into the sampled population following the strong population decline at approximately 14,500 cal. yrs BP. Although the star-like pattern of haplotypes at 11,500 cal. yrs BP is consistent with a signal of population expansion, with an estimated mutation rate of about 0.09 mutations/site/myrs, 3,000 yrs do not appear to be enough time to accumulate such a number of unique mutations. A potential immigration of individuals from adjacent areas carrying unique haplotypes probably explains the relapse in populations size around 11,500 cal. yrs BP. Analysis of mtDNA variation in modern and recent (up to 1,000 BP) populations on a broad geographic scale revealed a possible contact zone between regional phylogeographic groups at the Yangana-Pe-4 locality but provided no evidence for an effect of ongoing gene flow on genetic diversity in the modern sample from the main sampling locality at Pymva Shor. However, our simulation studies show that there are signs of ancient migration between Pymva Shor and adjacent regions. This finding is consistent with both the MCMC based analysis, which shows a fNe recovery following the bottleneck, and modern data suggesting higher migration rates in collared lemmings following a strong population density reduction [55]–[59].

Using ancient and modern DNA sampling we were able to show that small mammal demography can be highly dynamic. In the case of the collared lemmings we also found that this species drastically changed its migration pattern over time. Furthermore this study adds to the growing evidence that some populations of modern mammal species from the Arctic are deprived of genetic diversity, a pattern observed for such diverse species as polar bear [60], muskoxen [61] or wolf [62]. We found such a reduction of historical effective population size in an Arctic species to be a direct biotic response to warming climate. Although such a reaction may be expected given the ecological requirements of the collard lemming, it is interesting how exact the signal of climate warming is imprinted in the genetic diversity of this species. The close link between genetic diversity and fossil abundance also shows that the fossil record may indeed reflect species abundance rather than the vagaries of preservation and taphonomy.

The collared lemming, being adapted to arctic environments will most likely face a further demographic decline and range contraction or even extinction, when the average temperature continues to rise. Such a climate-driven demographic decline and range contraction of the collared lemming, which is a key member of northern communities, would have strong consequences for trophic interactions and ecosystem processes in the Arctic [11], [24]–[30]. Since collared lemmings are the main prey for four predators (the snowy owl, the Arctic fox, the long-tailed skua and the stoat), a strong climate-driven population decline in this species would most likely cause a severe reduction in predator populations and may lead to local extinction of the predators themselves [30]. Thus, a proper understanding of the effects and responses of this Arctic key species is crucial for predictions of possible future scenarios in the Arctic biota.

## Materials and Methods

### 0.1 Sampling and Radiocarbon Dates

We sampled 64 *Dicrostonyx torquatus* remains (left jaws) from Pymva-Shor (PS) in the northern Pre-Urals, for ancient DNA analyses. The layers of this site are dated to 21,910
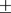
250 (L6 up), 13,090
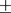
60 (L6 low), 10,000
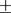
250 (L4) yrs BP and modern (surface). The radiocarbon dates for the different layers were taken from [23], (Table 1). We calibrated the dates using the Fairbanks0107 curve as provided online at http://www.radiocarbon.ldeo.columbia.edu/ and described in Fairbanks et al. 2005 [63]. Radiocarbon dates and calibrated radiocarbon dates are shown in Table S1.We collected 14 left jaws from the lower part of layer 6, 20 from the upper part of layer 6, 20 from layer 4 and 10 from the surface. A further 13 samples were collected from Yangana-Pe-4 (YP4). We included only samples from PS in the temporal demographic reconstructions to avoid bias in the results due to sampling of different populations. Our sampling of 10 to 20 individuals per time-point should provide enough power to detect a demographic change within the sampled period [64]. The samples from YP4 were solely used in additional analyzes to survey for evidence of gene flow.

### 0.2 DNA Extraction, Amplification and Sequencing

Extraction of the 77 left jaws (64 from PS and 13 from YP4) was performed following a silica-based extraction protocol after [65]. We used only left jaws for DNA extraction to rule out the possibility of sampling the same individual twice. About 100 mg to 150 mg of bone powder were used in each extraction, which was carried out in a clean-room environment built for handling ancient DNA to avoid external contamination from modern DNA and PCR products. All 77 extracted lemming remains yielded DNA of sufficient quality to amplify fragments of up to 152bp in length (including primers). Primers for use in the 2-step multiplex approach [66] and the 60 cycle PCR approach [67] (for replication of single missing fragments) were designed using the web-based tool primer3 [68]. Sequences of the primer oligonucleotides are provided in Table S2. Throughout the experiments we monitored for contamination using several controls (3 blanks for each extraction of 21 bones and 2 additional PCR blanks per primer pair). Each sequence position was determined from at least two independent amplifications, as misincorporations of nucleotides leading to C to T and G to A changes are very common in ancient DNA [69]–[72]. In rare cases of consistent differences between two independent amplifications we performed a third PCR to verify the actual nucleotide sequence. We used the direct-multiplex tagging protocol described in [73], but applied it to 2nd step - rather then 1st step amplicons - to create libraries for 454 FLX sequencing. All tagged libraries were quantified using the QPCR method described in [74] and sequenced on a 454 FLX platform (ROCHE). Single fragment amplicons (for missing replicates) were cloned using the Topo TA cloning Kit (Invitrogen, The Netherlands) and sequenced on an ABI 3730 capillary sequencer (Applied Biosystems, USA) [67]. As a result of 454 sequencing errors, we could not determine the exact length of two adenine homopolymer stretches within the 428 bp control region fragment, a well known problem of 454 sequencing [75], [76]. Therefore, we used the same length for the homopolymer sequence in all modern and ancient DNA sequences.

### 0.3 Data Analysis

The consensus sequences of the 64 samples from Pymva Shor (PS) were then aligned using the alignment editor BioEdit 7.0.8 [77] and transformed into a nexus file using the online converter available at http://www.hiv.lanl.gov/content/sequence/FORMAT_CONVERSION/form.html. We reconstructed statistical parsimony networks for each time-point (modern, 11,500 cal. yrs, 15,200 cal. yrs and 25,200 cal. yrs BP) separately using the software TCS 1.21 [78]. These networks were then connected to form a three dimensional network by combining haplotypes that were present at different time points. Furthermore, we reconstructed two additional parsimony networks using modern sequences from different regions surrounding PS (Pechora, Yamal and Taimyr, Figure 3A) and sequences from a second paleontological sampling site (Yangana-Pe-4, Figure 3B) to test for possible gene flow. All additional modern sequences were obtained from complete mitochondrial genomes sequenced from specimens from the University of Alaska Museum Mammal Collection, which will be published elsewhere (Fedorov *in prep*). The sequences used in this study can be accessed via GenBank (Accession numbers: HM008998-HM009023 and HM022324-HM022381), for an overview of the haplotype combinations see Table S3.

In order to test the possibility of sampling effects within our data set we used the software Bayesian Serial Simcoal (BayeSSC) [79] to simulate our temporal data under a constant population size model (fNe ranging from 30,000 to 50,000). We sampled 10 sequences for the modern, 20 for 11,500 cal. yrs BP, 20 for 15,200 cal. yrs BP and 14 for 25,200 cal. yrs BP time-points, respectively. Using average pairwise sequence difference (PD) as summary statistic reflecting the genetic diversity within our data we assessed the probability of sampling a similarly low genetic diversity by chance. We ran the simulation for 450,000 iterations. For each observed PD measurement we calculated its P-value using a one-sided test and set the significance level at 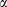 = 0.01. Bayesian Serial Simcoal is a modification of the program Simcoal 1.0 [80] which allows for temporal sampling.

#### 0.3.1 MCMC based Bayesian Inference

To infer demographic history directly from the data we used a Markov Chain Monte Carlo (MCMC) based Bayesian approach as implemented in the software BEAST 1.4.8 [35]. We used the software ModelTest 3.7 [81] to identify the best fitting nucleotide substitution model. The Akaike information criterion (AIC) supported the GTR+I model as best fitting model among those provided by BEAST 1.4.8 [35]. This nucleotide substitution model was used to estimate the genealogy best describing the data. The estimates of the female effective population sizes through time were obtained from the lineage coalescent rate. To smooth the variation of population size estimates over time and make the estimates less susceptible to outliers, several lineage coalescent events were summarized into groups. As the sensitivity of the analyses to pick up population size changes is depending on how many lineage coalescent events are summarized into groups we performed separate BEAST runs using 10 groups as default value and we chose group numbers on both sides of this value for our test (6 and 12, respectively). For each group number prior, three independent MCMC runs of 20,000,000 iterations each, sampling every 1000th step with a burn-in of 2,000,000 steps were performed. The program TRACER 1.4 [36] was used to verify the effective sample sizes (ESS) and the trace of the MCMC runs as well as to visualize demographic changes through time using a Bayesian Skyline Plot (BSP) [34]. To evaluate the support for the complex BSP prior compared with a simple constant size coalescent prior the *Dicrostonyx* genealogy was also reconstructed in BEAST 1.4.8 [35] under a constant size tree prior with a uniform prior for the population size (from 0 to 200,000). Posterior probabilities of the genealogies given our data under both population size priors were compared by estimating the log10 Bayes factor using TRACER 1.4 [36]. Furthermore we repeated the analyses under the BSP tree prior for 20 datasets with randomly shuffled sample ages to test if the estimated mutation rate does not solely depend on the age of the sample points.

#### 0.3.2 Approximate Bayesian Computation

We used the Approximate Bayesian Computation (ABC) approach [38] to test a number of population scenarios. The ABC analysis approximates the posterior distribution by using information from a prior distribution and extensive simulations rather than calculating the likelihood of the data directly. We used the software Bayesian Serial Simcoal (BayeSSC) [79] to simulate temporal data under a closed population-bottleneck model and an open population-bottleneck model including ancient migration. In order to compare both bottleneck models, prior knowledge of the bottleneck timing is crucial to distinguish models that do and do not implement ancient gene flow. We therefore carried out the ABC analysis of the closed single-population-bottleneck first. We then used the estimated bottleneck timing as a prior estimate in the open population-bottleneck model to infer the degree of historical gene flow that best fits the data. R 2.8.1 was then used to perform the rejection algorithm and the local linear regression adjustment and smooth weighting after Beaumont et al. 2002 [38]. In addition, we simulated our data under a single-constant-population size model to test whether the null hypothesis of a constant fNe over time can be rejected. We used uniform priors for the modern fNe, growth rate, event timing and severity, mutation rate and fNe before and after the bottleneck. Modern fNe ranges from 1 to 50,000 individuals and the new scaling factor for fNe after the bottleneck was specified as the fNe from 1 to 200,000 divided by fNe from 1 to 5,000 (fNe before and after the bottleneck, respectively). We simulated the data under possible values of the mutation rate ranging from 0.01 to 0.2 mutations/site/myrs and assumed an average generation time of one year for *Dicrostonyx torquatus* [44]. The software Treepuzzle 5.2 [82] was used to investigate the shape parameter of the gamma distribution of mutation rates (0.02 and 8). Since we merged sequences from CytB and CR we incorporated a Kimura 2-Parameter model to allow for heterogeneous mutation rate. Four sample groups were used to summarize the data. The same range of prior values for implied parameters was used in the constant size model. For the population fragmentation model (open population-bottleneck model) we simulated a split between 11,000 and 15,000 generations back in time into 4 populations. We used uniform priors for the modern fNe between 1 and 20,000. In order to simulate ancient migration, we specified a migration matrix with higher bidirectional migration between each adjacent region and PS and less direct migration between adjacent regions. For the higher migration we assumed a uniform prior of between 10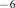 and 1 and for the lower migration between 10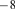 and 10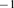. We assumed that the migration stopped between present and 1,000 generations back in time as there is no sign for recent migration in the modern data. We chose the number of segregating sites, average pairwise sequence difference and nucleotide diversity as summary statistics, because of their ability to unveil different aspects of population history [83].

We performed simulations for all models comprising 2,000,000 iterations. BayeSSC provides a separate output file containing all calculated summary statistics and parameter values. All required summary statistics of the empirical data were estimated using the software DNAsp v5 [84] and can be seen in Table 1. All R functions specific for the ABC approach are kindly provided by Mark Beaumont on his homepage (http://www.rubic.rdg.ac.uk/~mab/stuff/). We first applied the rejection algorithm to test the three models against each other as described in [39]. Local linear regression and smooth weighting was then applied using the function calmod(). This function estimates the posterior probability of a particular model using categorical regression.

In the next step we used rejection, local linear regression and smooth weighting to estimate the posterior probability distributions of the demographic parameters of the bottleneck model and the fragmentation model using the locfit function. We applied a tolerance of 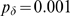 for parameter estimation and the model comparison approach, accepting the 5,000 and 6,000 closest estimates, respectively.

## Supporting Information

Figure S1 Parameter distribution of the average pairwise sequence differences (PD) at the differences. Black arrows represent the empirical estimates. Using P-values we show that low diversity within the modern and the 11,500 cal. yrs BP sampling points are unlikely to be an effect of biased sampling (P-values: Modern…0.00988; 11,500 cal. yrs BP… 0.00654; 15,200 cal. yrs BP… 0.18757 and 25,200 cal. yrs BP … 0.22578).
(0.36 MB EPS)

Figure S2 Marginal densities of the mtDNA substitution rate for the analysis using randomly shuffled dates. The distributions centered to the left close to a substitution rate of zero show the results for the randomly shuffled dates. The rate estimated for the original data set is given by the grey distribution with a mean of 9.6E-8.
(0.73 MB EPS)

Figure S3 Posterior probability distributions of different model parameters in the open population-bottleneck model. Density curves of the female population size for the four modern populations (Ne Population 1–4), the mutation rate (mutation rate), timing of the migration stop (migration stop) and the population split (split timing), the two migration rates between adjacent regions surrounding pymva shor (migration rate 1) and between less direct migration only between adjacent regions (migration rate 2). The x-axes are labeled according to the parameters they refer to.
(0.44 MB EPS)

Figure S4 Results of the model comparison approach using the rejection - and the regression algorithm after [39]. The open population-bottleneck model allowing for ancient migration (55.8%) and the closed population-bottleneck model (44.2%) are much stronger supported than the constant population size model (0%), thus, favoring a demographic reduction over a constant population size through time. A: Rejection Step, B: Regression Step.
(0.10 MB EPS)

Figure S5 Histograms of the summary statistics for the closed population-bottleneck model (A) and the open population-bottleneck model (B). Histograms of the eight summary statistics (Segsites…Segregating Sites and NucDiv…Nucleotide Diversity) used in the model comparison approach after [39] for the closed population-bottleneck model (A) and the open population-bottleneck model (B) to show the fit of the simulated data to the observed values. The green dotted-bashed line represent the empirical values.
(0.76 MB EPS)

Table S1 Sampling. All samples were taken from two sample sites (Pymva Shor and Yangana-Pe-4) in the Northern Ural, Russia. Radiocarbon dates and layer assignment according to [23] are shown as well as calibrated radiocarbon dates using the Fairbanks0107 curve [63].
(0.02 MB PDF)

Table S2 Primer-Oligonucleotide Sequences. Primers used in the 2-step multiplex approach [66] and the 60 cycle PCR approach [67].
(0.04 MB PDF)

Table S3 Haplotype composition of the Dicrostonyx torquatus samples used in the study. The first column shows the combination of the different haplotypes of the cytochrome B gene (C) and the control region (H). The second column shows the combined number of the specific haplotype combination, the following until the next to last refer to the time-points from Pymva Shor site and the last one refers to the samples from Yangana-Pe-4.
(0.04 MB PDF)
