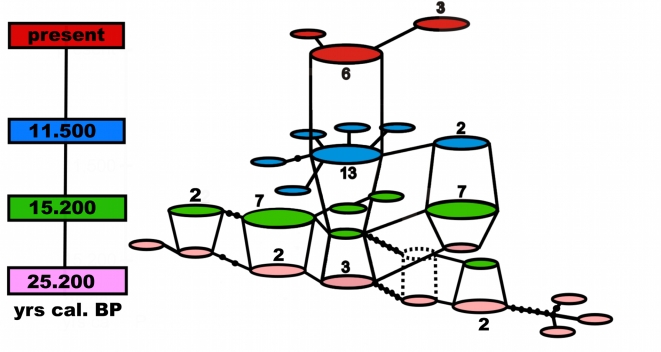# Correction: Influence of Climate Warming on Arctic Mammals? New Insights from Ancient DNA Studies of the Collared Lemming *Dicrostonyx torquatus*

**DOI:** 10.1371/annotation/25713b22-1ec4-4a23-86e2-f18873089157

**Published:** 2010-07-02

**Authors:** Stefan Prost, Nickolay Smirnov, Vadim B. Fedorov, Robert S. Sommer, Mathias Stiller, Doris Nagel, Michael Knapp, Michael Hofreiter

Figure 1 contains an error. Please view the corrected figure here: 

**Figure:**