# Supplementary material for: Influence of Climate Warming on Arctic Mammals? New Insights from Ancient DNA Studies of the Collared Lemming Dicrostonyx torquatus
Source: PLoS One. 2010 May 27;5(5):e10447. doi: 10.1371/journal.pone.0010447 (PMC2877706; doi:10.1371/journal.pone.0010447)
Supplement: Table S1 — Sampling. All samples were taken from two sample sites (Pymva Shor and Yangana-Pe-4) in the Northern Ural, Russia. Radiocarbon dates and layer assignment according to [23] are shown as well as calibrated radiocarbon dates using the Fairbanks0107 curve [63]. (0.02 MB PDF) [file pone.0010447.s006.pdf]

Name	Primer Sequence 5'-3'	Fragment length (including Primers)
H2Dt1F	GGATGCTATGACTCACCA	
H2Dt1R	TGGAATTATGTGGATGAATG	95bp
H2Dt2F	GTCTAGCTGGACTTATCATTCA	
H2Dt2R	AGTCCTTGAAGGTGGAATA	131bp
H2Dt3F	ATATAACAGTACTAATCATGCAAA	
H2Dt3R	GATAGGCGATTTCAGGT	148bp
H2Dt4F	AAAACAAGCTTAGCCAAA	
H2Dt4R	CCTGATACTGGTTTTTAAGTCA	142bp
H2Dt5F	CAGGACCTCTCATCCATT	
H2Dt5R	AAGCTACATCAACATTTATCA	152bp
CBDt1F	AGCAACAGCATTCTCGT	
CBDt1R	CCTCGTCCTACGTGTAAG	126bp
CBDt2F	GGCTAATTCGCTACATACA	
CBDt2R	ACAGCGAATAGGAGGATAAT	139bp
CBDt3F	GGCTCCTATAACATAATTGAA	
CBDt3R	AAGGATATTTGTCCTCATGG	107bp
CBDt4F	AGCAACAGCATTCATAGG	
CBDt4R	CTCAGATTCATTCTACTAGGG	125bp
