# Supplementary material for: Influence of Climate Warming on Arctic Mammals? New Insights from Ancient DNA Studies of the Collared Lemming Dicrostonyx torquatus
Source: PLoS One. 2010 May 27;5(5):e10447. doi: 10.1371/journal.pone.0010447 (PMC2877706; doi:10.1371/journal.pone.0010447)
Supplement: Table S2 — Primer-Oligonucleotide Sequences. Primers used in the 2-step multiplex approach [66] and the 60 cycle PCR approach [67]. (0.04 MB PDF) [file pone.0010447.s007.pdf]

**Locality**	**Layers**	**Age/Radiocarbon dates**	**Calibrated dates (Fairbanks0107)**
Pymva-Shor	surface	modern	-
Pymva-Shor	L4	10,000 ± 250 (GIN-9004)	11,538 ± 423
Pymva-Shor	L6 up	13,090 ± 60 (CAMS-38221)	15,246. ± 122
Pymva-Shor	L6 low	21,910 ± 250 (TUa-11501)	25,244 ± 359
Yangana-Pe-4	L2	up to 1,000	-
