# Supplementary material for: Influence of Climate Warming on Arctic Mammals? New Insights from Ancient DNA Studies of the Collared Lemming Dicrostonyx torquatus
Source: PLoS One. 2010 May 27;5(5):e10447. doi: 10.1371/journal.pone.0010447 (PMC2877706; doi:10.1371/journal.pone.0010447)
Supplement: Table S3 — Haplotype composition of the Dicrostonyx torquatus samples used in the study. The first column shows the combination of the different haplotypes of the cytochrome B gene (C) and the control region (H). The second column shows the combined number of the specific haplotype combination, the following until the next to last refer to the time-points from Pymva Shor site and the last one refers to the samples from Yangana-Pe-4. (0.04 MB PDF) [file pone.0010447.s008.pdf]

	combined	Modern	11,500 cal. yrs BP	15,200 cal. yrs BP	25,200 cal. yrs BP	1000 BP
C1H1	33	6	13	1	3	10
C1H2	1	1				
C1H4	1		1			
C2H1	1	1				
C3H1	2	2				
C4H1	2					2
C4H3	1					1
C5H1	10		2	7	1	
C6H1	1		1			
C7H1	1		1			
C8H1	1		1			
C9H1	1		1			
C10H5	1			1		
C11H6	3			2	1	
C11H9	1				1	
C12H7	3			1	2	
C12H12	1				1	
C13H1	9			7	2	
C13H5	1			1		
C14H8	1				1	
C14H11	1				1	
C15H10	1				1	
$\sum$	77	10	20	20	14	13
